# Development of an antimicrobial inorganic polymer based on fly ash and metakaolin incorporated by nano-TiO_2_ for reactive dye removal

**DOI:** 10.1038/s41598-023-47032-9

**Published:** 2023-11-14

**Authors:** Doaa A. Ahmed, Morsy A. El-Apasery, Shereen M. Ragai

**Affiliations:** 1 Chemistry Department, Faculty of Women for Arts, Science and Education, Ain Shams University, Cairo, 11757 Egypt; 2 Dyeing, Printing and Textile Auxiliaries Department, Textile Research and Technology Institute, National Research Centre, 33 El Buhouth St, Cairo, 12622 Egypt

**Keywords:** Environmental sciences, Chemistry, Materials science, Nanoscience and technology

## Abstract

Advanced and eco-friendly construction materials are being developed to reduce pollution and improve wastewater treatment efficiency. One such material is a photocatalytic nanocomposite that uses industrial wastes and natural substances to eliminate pollution. A recent study explored using an inorganic polymer composite (FM) made from a mixture of 70% fly ash and 30% metakaolin, with sodium hydroxide and sodium silicate as an alkali activator. The study evaluated the mechanical and hydration characteristics of the FM composite after 28 days in 100% humidity at room temperature. The study also examined the effect of adding 2.5 wt.% of Nano-TiO_2_ to FM composite and how it affects its properties. Results indicate that adding Nano-TiO_2_ to FM composite enhances its mechanical, antibacterial, and photocatalytic capabilities. Specifically, FM-TiO_2_ composite showed 90% removal of reactive blue 19 dye effluent in sunlight after 90 min, making it an excellent choice for sustainable wastewater treatment. This study presents a cost-effective, eco-friendly solution to wastewater treatment, with added antimicrobial properties from Nano-TiO_2_.

## Introduction

The advancement of society is closely linked to the progress in the industrial sector. However, the industrial sector, which consumes significant water, has caused environmental damage through resource depletion, pollution, and solid waste generation^1,2^. The rise in synthetic dyes in effluents from various industries has led to environmental issues such as water body impairment, hindered light and oxygen penetration, and toxic effects on aquatic biota^3^. Synthetic dyes, particularly water-soluble reactive and acid dyes, are a major cause of environmental pollution due to their resistance to conventional wastewater treatment. Their widespread use, low synthesis costs, and diverse range of colors make them a significant concern^4^. However, their poor biodegradability means that residual colors can persist in wastewater streams, posing a threat to aquatic life due to high levels of chemical and biological oxygen demand, toxic, allergenic, skin-irritant, mutagenic, and carcinogenic characteristics^5,6^.

Reactive dyes, such as Reactive Blue 19 (RB19), are widely used in the textile industry due to their high solubility and ability to form covalent bonds^7^. However, around 12–15% of the dye remains unreacted, leading to elevated concentrations in textile wastewater^8^. Reactive Blue 19 (RB19) is a type of anthraquinone dye that has been extensively utilized in various industries such as textiles, food, paper, pharmaceutics, and cosmetics. According to Holkar et al.^9^ it has been approximated that approximately 10–15% of the unused dye is ultimately discharged into water bodies during the dyeing process. The removal of RB19 from wastewater has garnered significant attention due to its high toxicity and stability, which results in severe environmental contamination. In recent times, a range of techniques have been utilized to treat wastewater originating from textile production. These include the employment of conventional methods such as coagulation^10^, adsorption^11^, aerobic or anaerobic biosorption^12^ and advanced chemical oxidation or photochemical degradation^13^. Photochemical degradation is widely considered to be one of the most efficient methodologies available.

Photochemical degradation, a process that employs various semiconductor oxides, namely titanium dioxide (TiO_2_), tungsten trioxide (WO_3_), tin dioxide (SnO_2_), zinc oxide (ZnO), combined oxides^14,15^, and modified oxides^16^, is known for its photocatalytic activity. Of these, TiO_2_ has been widely utilized due to its unique properties, including non-toxicity, low cost, potent oxidation capabilities, and exceptional chemical stability^17^. One of the Limitations inherent in this process pertains to the arduousness of segregating the liquid and solid photocatalyst subsequent to the elimination of contaminants. As a result of this challenge, numerous solid substrates^18,19^ have been examined by researchers as a means of immobilizing WO_3_ and TiO_2_^20^.

On the other side, the matter of waste deposition in industrial facilities warrants consideration, specifically in relation to the disposal of fly ash (FA) that results from power plant operations. The ingredients of fly ash vary considerably based on the origin and composition of the coal employed during combustion. However, all fly ash contains substantial amounts of silicon dioxide (SiO_2_), aluminum oxide (Al_2_O_3_), iron oxide (Fe_2_O_3_), and calcium oxide (CaO)^21^. The utilization of fly ash waste in a contextualized methodology could potentially provide efficacious remedies for eliminating the detrimental impact of industrial dyes on the environment. The chemical composition of manufacturing dyes, which incorporates one or more aromatic rings with chromophore groups such as the azo group (–N = N–), renders them highly stable and resistant to degradation^22^.

Several studies have been conducted to enhance the mechanical characteristics of photocatalyst materials while minimizing the amount of material utilized. Numerous studies have been undertaken in order to achieve this goal using various structural bases, such as cementitious materials, clays, and geopolymers, for photocatalysts^23^. Geopolymers are synthesized by means of geopolymerization, a chemical process that entails the interaction between aluminosilicate and an alkaline solution generally comprising sodium hydroxide and sodium silicate^24^. Due to their amorphous polymeric structures, geopolymers exhibit qualities such reduced carbon dioxide emissions during manufacture, excellent adsorption ability, high stability chemically, strong compressive strength, high durability, mechanical resistance, and thermally stable. Geopolymers are being used as "self-cleaning" building materials, which is another new application for them, they can also be used as supports for photocatalysts^25^. The raw materials used for the manufacturing of geopolymers, regardless of their intended use, demand a considerable amount of silica and alumina, which can be obtained from a range of sources such as fly ash, kaolin, zeolites, rice hulls, etc.

Various studies have revealed that geopolymers produced from diverse raw materials possess the potential to serve as adsorbents and/or photocatalysts, as noted by Ahmed et al.^11^, Fallah et al.^26^, and Hertel et al.^27^. Prospectively, adding semiconductor-based photocatalysts to geopolymers may enhance their capacity for self-cleaning^28,29^. On conventional building materials, this strategy has been demonstrated to be effective^30,31^ For instance, Janus et al.^32^ showed that the inclusion of nitrogen and carbon co-modified TiO_2_ photocatalysts into raw cement material improved the removal of a typical organic water contaminant by up to 60% under UV/vis light. Kaya-Özkiper et al.^33^ investigated the performance of red mud (RM) and metakaolin (MK)-based geopolymers for the adsorption and photocatalytic degradation of methylene blue to demonstrate their potential as self-cleaning, environmentally friendly construction materials.

On the other hands, geopolymers and OPC-based concrete have antimicrobial properties in dry conditions due to their alkaline nature. However, certain bacteria, like sulphur-oxidizing bacteria, can inhabit these surfaces in humid conditions, producing acidic compounds that damage the material and lower its pH^34^. The porous nature of geopolymers makes them more vulnerable to microbial corrosion^34^. High resistance to microbial degradation is crucial for construction materials in aggressive environments like sewers, sewage treatment plants, and water. Geopolymer and OPC-based concrete antimicrobial protection techniques have been developed to reduce microbial deterioration and use in aggressive applications like sewage systems. Inorganic agents like copper oxide and zinc oxide can combat microorganisms in nanoparticles but may pose toxicity or environmental hazards. TiO_2_, due to its low toxicity and photocatalytic activity, can serve as an antibacterial and self-cleaning ingredient in outdoor geopolymer applications due to its low toxicity^34^.

There is a lot of research in the literature on the influence of nanoparticles on the photocatalytic capabilities of geopolymers toward various types of organic dye. On the other hand, the effect of geopolymer-containing nanoparticles on the elimination of reactive organic dyes pollutant has received little attention. The goal of our present study is to create a novel inorganic polymer (geopolymer) that can be made from industrial and natural waste materials such as fly ash and metakaolin, has superior cement properties, and has environmental and biological applications. Our study focuses on the effect of the addition of 2.5% by weight of nano TiO_2_ on the mechanical and hydration properties of a geopolymer composite containing 70% FA and 30% MK (FM). In the same manner, we study the photocatalytic behavior of FM and FM-TiO_2_ geopolymer composites for the removal of reactive organic dye pollutants (Reactive blue 19) from wastewater under sun light. Additionally, enhancing the novel low-cost geopolymer composite's (FM) antimicrobial properties with the addition of nano-TiO_2_ particles.

## Materials and research methodologies

### Materials

**Figure 1:**
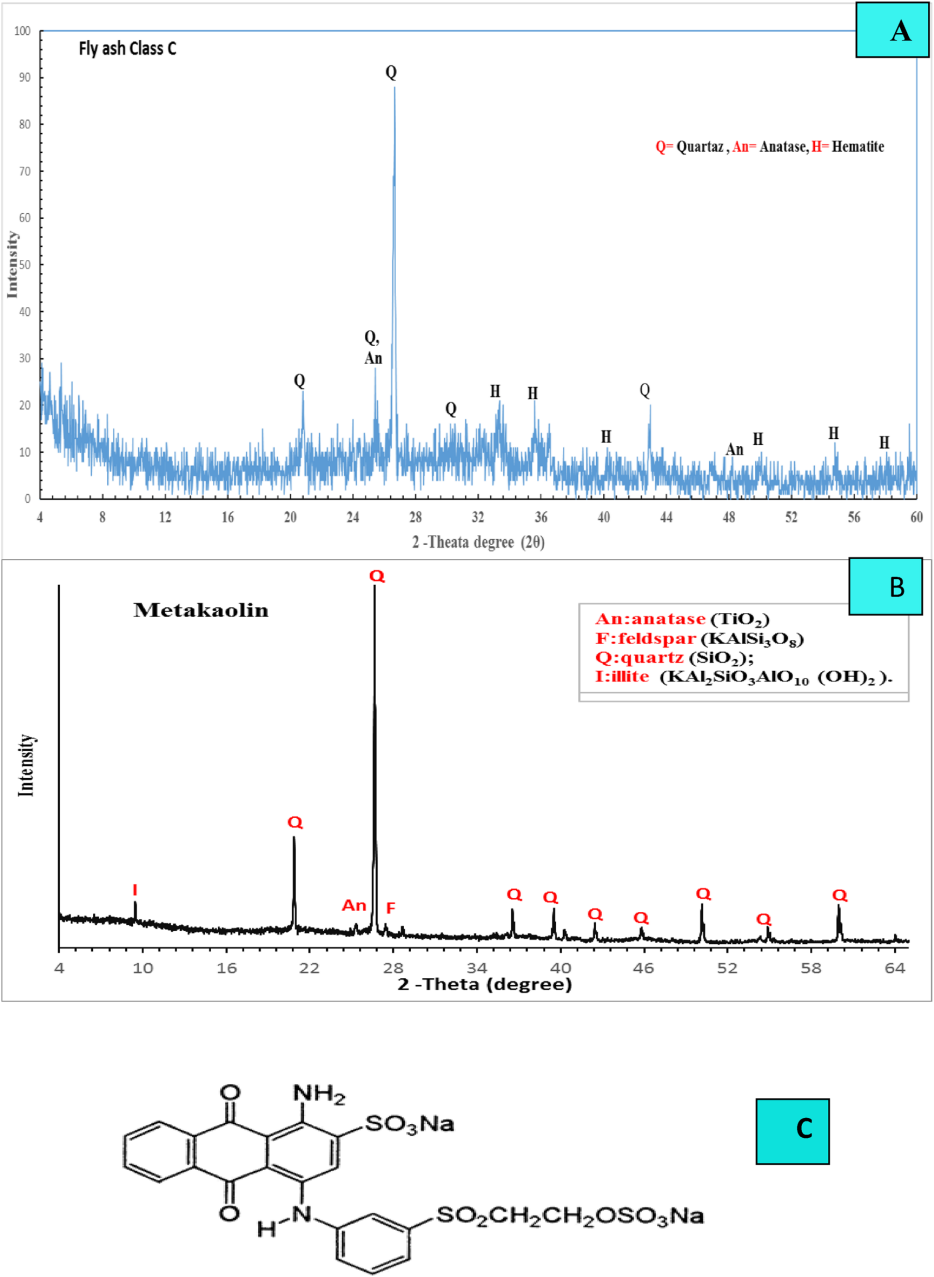
XRD patterns for the raw materials (**A**, **B**) and Chemical Structure of Reactive blue 19 (**C**).

Fly ash (FA), metakaolin (MK), sodium hydroxide (NaOH), and liquid sodium silicate (Na_2_SiO_3_) were the ingredients employed in this work to manufacture the fly ash/metakaolin-based geopolymer (FM). Furthermore, the nano titanium oxide used in this research is Titanium (IV) oxide TiO_2_ NPs Nano powder, 21 nm primary particle size (Tem), ≥ 99.5% trace metals basis, Lot # MKBV3126V was purchased as 718,467-100G from ALDRICH Chemistry^35^. Hemts Construction Chemical Company in Cairo, Egypt, provided the metakaolin for the examination. Cairo, Egypt's El Nile overseas chemical company offers Class C fly ash (FA). From Burg Al-Arab, Alexandria, Egypt, Silica Egypt Company has supplied commercial liquid sodium silicate (LSS) with a silica modulus of 2.80 SiO_2_/Na_2_O.Silica. The NaOH flakes have a purity of 99% and are purchased from EL-Goumhouria Chemical Company in Cairo, Egypt. Figure 1A,B illustrate the XRD patterns of fly ash and metakaolin. Table 1 additionally demonstrates the raw material's chemical oxide composition, which was utilized in the current study. The adsorption and photo-degradation experiments were conducted using the effluent from a reactive blue 19 dyeing bath. Figure 1C depicts the structure of this dye.

**Table 1:** XRF analysis of the chemical oxide composition of the raw materials, in mass%.

Chemical composition	FA	MK	LSS
SiO_2_	38.16	64.86	32.8
Fe_2_O_3_	14.90	0.55	–
CaO	18.10	0.52	–
Al_2_O_3_	17.20	30.10	–
SO_3_	1.17	0.14	–
Na_2_O	1.18	0.15	11.7
Cl^-^	0.06	–	–
MgO	4.46	–	–
TiO_2_	1.09	2.72	–
K_2_O	1.84	–	–
P_2_O_5_	0.25	–	–
MnO	0.21	–	–
BaO	0.11	–	–
SrO	0.11	–	–
CuO	0.02	–	–
ZrO_2_	0.02	–	–
Tl_2_O_3_	0.02	–	–
LOI	0.24	0.93	–
H_2_O	–	–	55.5
Total	99.99	99.97	100

*MK* metakaolin, *FA* fly ash, *LSS* liquid sodium silicate.

### Methods

#### Dyeing procedures

The wool was first dyed at a temperature of 95 °C and a pH of 4.5 for an hour. The wastewater from this dyeing process was collected. Afterwards, the same wastewater was re-used to dye the wool again for another hour at 95 °C and pH of 4.5. The wastewater from the second dyeing process contained dye content of 500 mg/L and was treated for further use.

#### Preparation of eco-friendly inorganic polymer composite samples

*Preparation of FA/MK geopolymer composites for mechanical and microstructure, and antibacterial examination.* For the manufacture of geopolymer pastes FM and FM-TiO_2_, 70% fly ash (FA) is first thoroughly mixed with 30% metakaolin (MK), in a dry environment until entirely homogenous. In the case of geopolymer mix F, we use 100% fly ash to prepare the paste. The alkaline activator solution was created by combining sodium hydroxide pellets with liquid sodium silicate in a 15:15 wt.% solid ratio. The liquid was allowed to cool to room temperature after creating a clear gel^36^. In the case of geopolymer mix FM-TiO_2_, we add 2.5 wt.% from nano TiO_2_ to alkaline activator solution and stir well for half an hour. To create a homogeneous geopolymer paste, dry components, and an alkaline activator solution are mixed with a total liquid/solid ratio of 0.27. Table 2 summarizes the components of each mix, in addition to the liquid/solid ratio that gives standard consistency. Next, one-inch-diameter cubic stainless-steel molds are filled with freshly developed pastes. To achieve the process of setting and hardening, the molds are kept at room temperature for the first 24 h with a relative humidity of 100%. The cubes were de-molded after molding and covered and cured for 3.7, 14, and 28 days at 100% relative humidity. The cubes were removed from their curing environment after each hydration period, and the compressive strength and total porosity% of various geopolymer composite mixes were measured. For the antibacterial test, six-disc specimens with a diameter of 12 mm and a thickness of 2 mm were created for each geopolymer composite (FM and FM-TiO_2_). These specimens underwent 7 days of hydration under curing conditions of room temperature and relative humidity of 80–90%.

**Table 2:** The chemical composition of the tested geopolymer mixes and their liquid/solid (L/S) ratio.

Mix name	FA %	MK %	Nano TiO_2_ wt.%	NaOH wt.%	Na_2_SiO_3_ wt.%	L/S ratio
FM	70	30	–	15	15	0.27
FM-TiO_2_	68.25	29.25	2.5	15	15	0.27

*Preparation of adsorbent samples from fly ash/metakaolin based -geopolymer composite.* After 7 days of curing for geopolymer mix FM and FM-TiO_2_, the broken compressive strength test cubes were employed for adsorption and photodegradation examinations. These broken cubes were then immersed in a 1:1 mixture of ethanol and acetone to stop the hydration process. The mixture was agitated for 30 min on a magnetic electrical stirrer, after which the residue was filtered, washed in ethanol, and dried for 24 h at 50 °C^37^. After being ground to a mean particle size of 100 μm, dried samples are put in a desiccator. Table 2 provides the composition of the various mixtures in addition to the liquid/solid ratio that produced homogenous consistency.

### Investigation techniques

#### The mechanical properties of fly ash/metakaolin-based geopolymer composite with and without Nano TiO_2_.

The compressive strength of the hardened paste is determined using a set of three cubes. The three measurements are averaged out to get the data, which is shown in MPa. The compressive strength was evaluated in this study utilizing a manually operated compression device (D550-control type, Milano, Italy). The hydration of crushed cubic specimens is stopped after the compressive strength test. Before being preserved for morphological examination, the crushed samples were mixed with an alcohol and acetone stop solution (1:1) and dried at 50 °C for 24 h^37^. On the other hand, the total porosity% of a geopolymer mixes composite is estimated by weighing specimens of the dry paste suspended in air and water, labeled W1 and W2, for three separate cubes. The weight of these cubes in the air, known as W3, is then calculated after they have dried at 100 °C for around 24 h. The following formula (1) is used for the calculation of the percentage of total porosity (%P)^38,39^.

$$ {\text{Total }}\;{\text{porosity = }}\frac{{{\text{(Saturated }}\;{\text{weight - dried }}\;{\text{weight)}}}}{{{\text{(Saturated }}\;{\text{weight - suspended }}\;{\text{weight)}}}} \times {100} $$

1 $$ {\text{P}}\% \, = \, \left[ {\left( {{\text{W2 }} - {\text{ W1}}} \right) \, / \, \left( {{\text{W2 }} - {\text{ W3}}} \right)} \right] \, \times { 1}00 $$

#### The microstructure Analysis (XRD, SEM and FTIR).

A complete investigation of the phase composition of the hydration products in several samples of geopolymer mix was made possible by the X-ray diffraction analysis carried out on the Bruker D8 Discover diffractometer (Germany). Cu-K radiation with a wavelength of 1.45 A^o^, a pixel detector set to 40 kV, and 40 mA was used in conjunction with a Ni-filtered diffractometer to get precise findings. Important details on the composition, characteristics, and degree of amorphousness of the geopolymer mix samples were disclosed by the data gathered from this analysis. On the other hand, FTIR measurements were performed on a Perkin Elmer 1430 infrared spectrophotometer (USA) using pellets of potassium bromide (KBr), with wave numbers ranging from 400 to 4000 cm^−1^. The functional groups of the hydration products created for various geopolymer composite samples were determined using this analytical technique. High-resolution scanning electron microscopy (SEM) was used to characterize the surface and structural morphology of the geopolymer samples produced. Analysis studies were conducted using FEI Quanta FEG 250 equipment (USA).

#### Adsorption experiments

On the adsorption capabilities of the FM geopolymer composite against reactive blue 19 dyeing bath effluent, the impacts of the addition of nano TiO_2_, adsorbent dose, pH value, and contact time were examined. A certain amount of the adsorbent (0.01–0.15 g) and 50 mL of dyeing bath effluent solution with a concentration of 250 mg/L were combined in a water bath at 140 rpm and 30 °C at various contact times and pH levels ^40–42^. Reactive blue 19 dyeing bath effluent was tested to determine the optimum pH, contact time, and adsorbent dosage that would provide the highest removal efficiency. Filtration could be used to establish a residual of the sample solutions. Using V-670 Spectrophotometer, we measured the absorbance [at max = 595 nm for reactive blue 19 dye] and then deduced the solution's concentration from the calibration curve. The Removal effectiveness % was calculated using Eqs. (2) and (3), which showed how much dye had been adsorbed onto the geopolymer material^43,44^.

2 $$ {\text{q}}_{{\text{e}}} = \left( {{\text{ C}}_{{\text{o}}} - {\text{C}} } \right) {\text{V}}/{\text{W}} $$

While C_o_ and C indicate initial and equilibrium liquid-phase concentrations in milligrams per liter, respectively; V represents the volume of the solution in milliliter, and W indicates the weight of the adsorbent in grams.

3 $$ {\mathbf{Removal}}\; \, {\mathbf{efficiency}} \, \% \, = {\mathbf{100}} \, \left( { \, {\mathbf{q}}_{{\mathbf{e}}} / \, {\mathbf{C}}_{{\mathbf{o}}} } \right) $$

#### Photodegradation experiment

In order to examine photodegradation, 0.1 g of the geopolymer composites (FM and FM-TiO_2_) were mixed with 50 mL of a solution containing 50 mg/L of reactive blue 19 dye (RB) effluent and put in a quartz beaker under magnetic stirring. For different periods of time—30, 90, 120, 180, and 240 min—the system was directly exposed to sunlight, a natural source of UV radiation. A residual of the sample solution after each time interval can be obtained via filtration. A Jasco V-670 double-beam spectrophotometer (Hachioji Tokyo, Japan) was used to evaluate the RB dye effluent content in the solution via absorbance at 593 nm (λ-max of RB) in the UV–vis spectrum in order to analyze the decomposition of organic dye effluent. The Removal effectiveness% was calculated using Eqs. (4).

4 $$ {\mathbf{Removal}}\; \, {\mathbf{efficiency}} \, \% \, = \left( { \, {\mathbf{C}}_{{\mathbf{o}}} - {\mathbf{C}}_{{\mathbf{t}}} } \right)/ \, {\mathbf{C}}_{{\mathbf{o}}} \times \, {\mathbf{100}} $$

C_o_ and C_t_ denote the dye solution's concentration at the start and end of the absorption process, respectively.

#### Antibacterial activities test of the prepared eco-friendly inorganic polymer composites with and without nano-TiO_2_

Six pathogenic bacterial strains representing Gramme-positive and Gramme-negative bacteria, including Staphylococcus aureus, Escherichia coli, Bacillus subtilis, Candid, Aspergillus, and Pseudomonas, were used to test the antibacterial activity of two geopolymer composites FM and FM-TiO_2_. To conduct the antimicrobial susceptibility test, the Kirby-Bauer Disc Susceptibility Test and measuring zones of inhibition methods were utilized^45,46^. To grow the bacteria, we used MacConkey agar, MacConkey broth, and nutrition agar as the growth media. The manufacturer's instructions were followed in the preparation of all the media. We created an inoculum for each bacterial isolate by emulsifying 2–4 colonies in sterile distilled water and adjusting the turbidity to 1.5 × 108 CFU mL^−1^ (similar to 0.5 McFarland standard) using the Koneman et al.^47^ method. We then applied the standardized bacterial suspension to the agar plates using a sterile cotton swab. The plates were left to dry for 2–5 min. After that, two geopolymer composite discs (FM and FM-TiO_2_) were put on the plates and gently pushed to make sure the agar was adequately covered. To prevent inhibition zones from overlapping, a minimum gap of 12 mm between the plates' borders was maintained. For two to five days, the plates were incubated at 37 °C. Following that, the plates were investigated and the diameters of the inhibiting zones in millimetres (mm) were measured, and this information was used to determine the test agent's antibacterial activity. The experiment was repeated several times, and the average reading was noted.

## Results and discussion

### The mechanical and microstructure characteristics of eco-friendly inorganic polymer composite with and without nano-TiO_2_

#### Compressive strength evaluation

**Figure 2:**
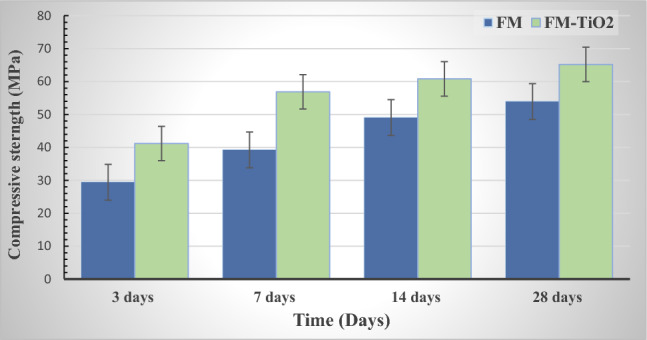
Compressive strength data for inorganic polymer composite (FM) with and without nano-TiO_2_ throughout a range of hydration times.

Compressive strength experiments were conducted to examine the mechanical characteristics of inorganic polymer (geopolymer) composites incorporating and without nano-titanium oxide (2.5 wt%) (FM and FM-TiO_2_) (Fig. 2). As illustrated in Fig. 2, all geopolymer mixes possess compressive strength values rising as the cure period increases. This is explained by the fact that a high alkali medium accelerates fly ash and metakaolin dissolution, providing the system with silicate and aluminate species. As a result, this led to an increase in the rate of hydration as well as the production of extra hydration products like CSH, CASH, and N-(C)ASH gel that precipitated in the open pores, enhancing the compressive strength^13,48^. In comparison to pure geopolymer composite (FM), geopolymers with nano-TiO_2_ added (FM-TiO_2_) demonstrated greater mechanical strength. After 28 days of hydration and during the extended hydration period, the incorporation of nano-TiO_2_ accelerated the polymerization process and enhanced the compressive strength of the composite^49^. The geopolymer composite MF-TiO_2_ attained its maximum compressive strength value (65 MPa) after 28 days of hydration. It concluded that the interfacial transition zone was reduced as a result of the interfacial adhesion between the nano-TiO_2_ particles and the fly ash/metakolin-based geopolymer^50^. The impact of nanotitanium oxide on the mechanical attributes of geopolymers has been the subject of numerous research reports^51–53^. In our study, the compressive strength enhanced by the addition of 2.5 wt.% of nano-TiO_2_ (FM-TiO_2_) was 17.65% higher than that of the geopolymer sample FM after 7 days of hydration. On the other hand, according to our results, the compressive strength improved by 10.96, 11.76 and 11.28% compared to the MF sample at different hydration periods of 3, 14, and 28 days, respectively. Our findings are consistent with later studies^53,54^, which reported that the incorporation of nano-titanium dioxide (NT) accelerated the polymerization process and enhanced the compressive strength of geopolymer composites.

#### Total porosity (P%) examination

**Figure 3:**
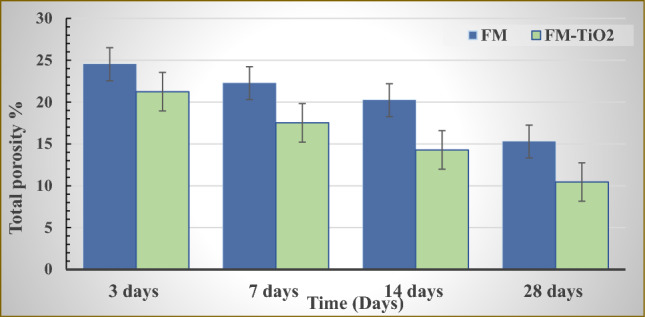
Total porosity % results for inorganic polymer composites (FM) with and without nano-TiO_2_ throughout a range of hydration times.

The total porosity of several geopolymer mix cubes FM, and FM-TiO_2_, cured at 100% humidity for 3, 7, 14, and 28 days are displayed in Fig. 3. The total porosity % values of all inorganic polymer composites (FM, and FM-TiO_2_) decreases with increasing curing time. These data show how the polymerization process is developing by time^11,39^. There was an increase in the production of hydration products, which precipitated in certain open, accessible pores and decreased the percentage of total porosity^11^. As illustrated in Fig. 3, adding nano-TiO_2_ to geopolymer composite MF decreases the total porosity values at all hydration stages. This may be caused by a reduction in the number of pores as a result of the inter-particle diffusion of nano-TiO_2_ into the interior pores of the geopolymer matrix^39^, which causes the filling of open pores and lowers the overall porosity values.

#### Fourier transform infrared spectroscopy analysis (FTIR)

**Figure 4:**
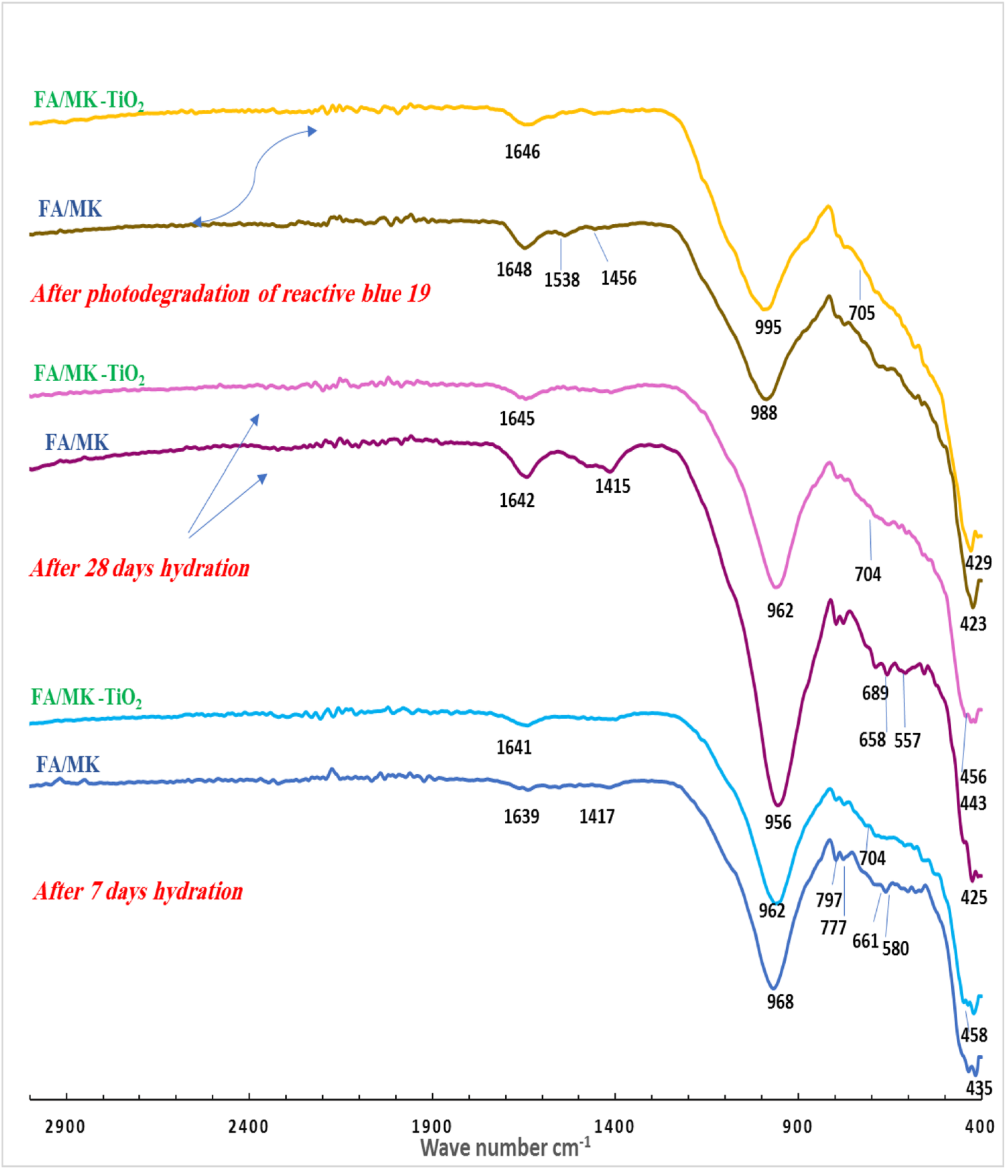
FTIR spectra of fly ash/metakaolin-based geopolymer composites (FM, and FM-TiO_2_) at 100% humidity for 7 and 28 days, and after the sunlight photodegradation process.

The Fig. 4 shows the Fourier transform infrared (FTIR) examination results of fly ash and metakaolin-based geopolymer pastes (MF and MF-TiO_2_) after 7 and 28 days of hydration. In contrast, Fig. 4 also shows the FTIR of the remaining precipitate of the geopolymer composites following the photocatalytic degradation process of reactive blue 19 under sunlight. As we notice from Fig. 4, the absorption band in the range of 1415–1456 cm^−1^ was formed as a result of the carbonation of calcium hydroxide. It appeared in the FTIR spectrum of the FM composite and completely disappeared in the FM-TiO_2_ mix, which may be attributed to the inter diffusion of NT particles inside open pores of geopolymer composites and inhibited the carbonation of hydration products^49^. For both geopolymer composites, the FTIR spectra show absorption bands in the ranges of 1639–1648 cm^−1^ that are connected to the bending vibration modes of the H–O–H group. It demonstrates the way fly ash and metakaolin species may decompose and condensation when an alkaline activator solution is used, producing an excessive quantity of hydration products inside the geopolymer matrix^55^. The inclusion of NT had no noticeable effect on the type of hydration products formed in FTIR spectra, as we notice from Fig. 4. A prominent absorption band between 956 and 968 cm^−1^ is also seen in Fig. 4 for both FM and FM-TiO_2_ composites, and it is thought that this band is related to the asymmetric stretching vibrations of Si–O–T (where T is either Si or Al). This is supported by the intensity of bands at around 777 cm^−1^ and 689 cm^−1^, which, respectively, correlate to the symmetric stretching vibrations of (Si–O–Si) and (Si–O–Si or Al–O–Si), confirming the complete dissolution of unreacted silica and the progression of the polymerization process^38,39^. As we observed from Fig. 4 the incorporation of NT inside the geopolymer retards the hydration process with time, as indicated by a decrease in the intensity of the asymmetric stretching vibrations of Si–O–T at 962 cm^−1^ for the FM-TiO_2_ mix after 28 days compared with the reference sample FM. These may be related to the presence of NT decreasing the number of pores on the surface of the geopolymer composite, which affects the geopolymerization process^49^. Furthermore, the presence of Ti–O–Ti photosensitive species was linked to the band's appearance at approximately 704 cm^−1^ in FM-TiO_2_ geopolymer^49^. Another indicator of NT particle dispersion inside the geopolymer matrix was the band of the O–Si–O bending vibration appearing for FM-TiO_2_ at 453–458 cm^−1^^49,56^. On the other hand, Fig. 4 shows that after degradation by sunlight, the asymmetric stretching vibration of the Si–O–T band in the FM and FM-TiO_2_ composites containing reactive blue 19 dye effluent shifts to higher wave numbers of 988 and 995 cm^−1^, respectively. This could be the result of the dye molecules adhering to the geopolymer matrix's active site, slowing down the rate of geopolymerization ^11,39^.

#### Crystalline phase identification (XRD)

**Figure 5:**
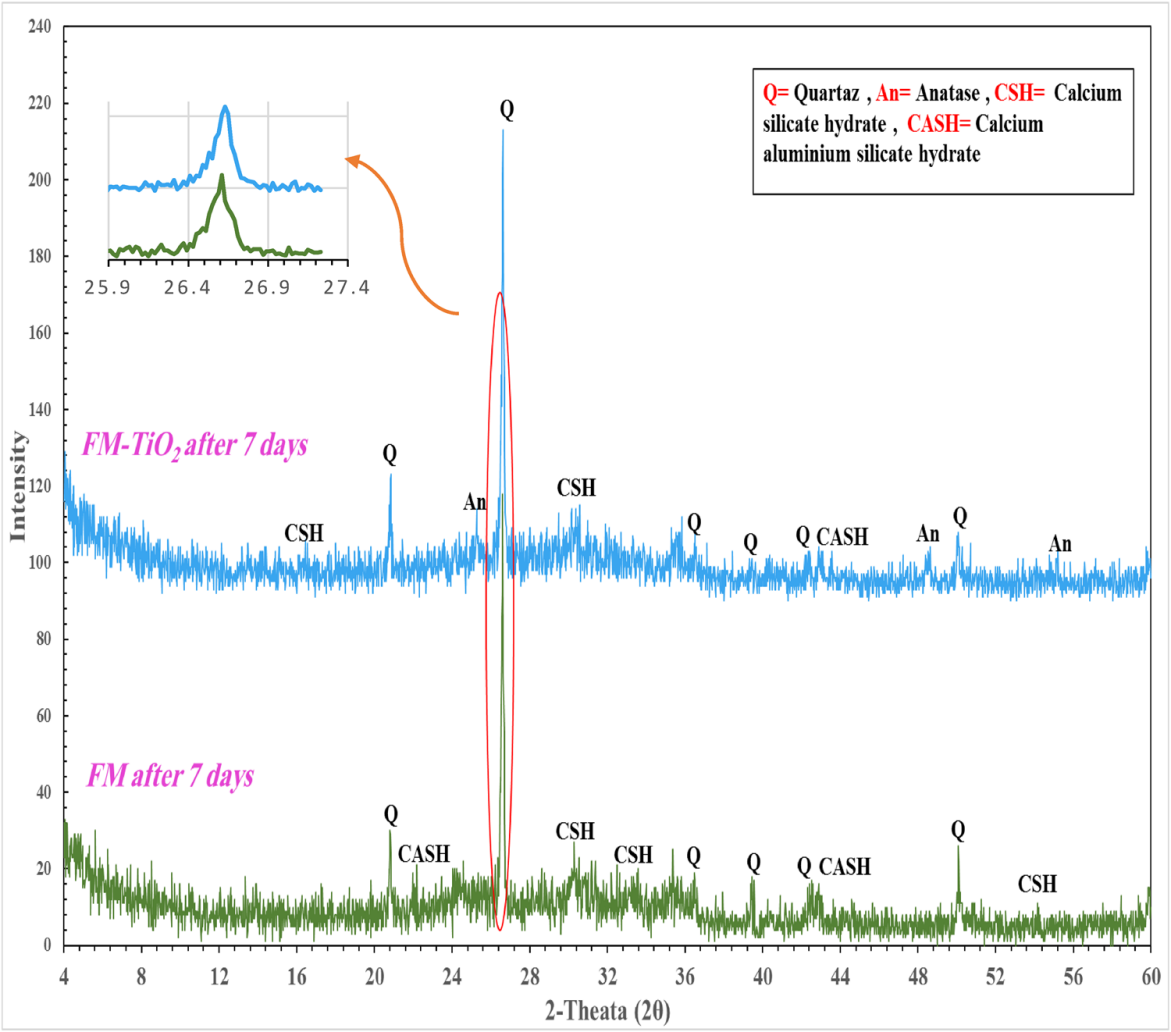
X-ray diffraction pattern of geopolymer composites with and without with various nano-TiO_2_ after 7 days of hydration.

After 7 days of being hydrated, the XRD patterns of FM and FM-TiO_2_ geopolymer composites are displayed in Fig. 5. The fly ash and metakaolin species seem to have completely disintegrated and dissolved due to alkaline activation and the creation of an amorphous geopolymer matrix, according to Fig. 5, which displays a hug and broad hump between 2θ = 22°–38°^48,49^. The study found that two types of hydration products were formed during the geopolymerization process. The first type is a crystalline phase, verified as C–(A–S–H) with d-values of 3.70, 3.29, and 2.61 Å (Fig. 5). The second type of hydration product was the amorphous to semi-crystalline phase C-S–H. This was evident in both geopolymer composites' XRD patterns at 2θ = 17°, 29°, 33°, and 54°, indicating the generation of amorphous and crystalline phases throughout the geopolymerization process. The addition of NT particles to geopolymer composite FM has no effect on the type of hydration product created^49^, as shown in Fig. 5. The XRD spectra also reveal strong peaks at 26.8°(2θ) (quartz peak), indicating that the geopolymer composites have formed a glassy phase^50^. The Fig. 5 illustrates the effect of adding nano-TiO_2_ filler to the geopolymer composites FM, showing a slight shift to a higher angle (from 26.58⟶26.63°). This indicates the filler's destructive effect on the ordered arrangement of the geopolymer side chains and the consequent enhancement of the amorphous phase^50^. The interaction between the filler surface and the geopolymer may be what is causing this detrimental impact^49,50^. We also observed that when NT was added to the geopolymer composites, the anatase phase developed at 2θ = 25.25° and 48°^49^. Additionally, we noticed a reduction in the intensity of quartz peaks, indicating that NT accelerates saturation and creates saturated materials through polymerization^49^. As a result, the size and quantity of unreacted raw material particles were thought to be reduced. This XRD study's observations show a harmonious correlation with Raj et al.'s 2023 findings^49^. The addition of NT improved the properties of fresh and hardened geopolymer composites.

#### Scanning electron microscope (SEM) of different eco-friendly geopolymer composite mixes

**Figure 6:**
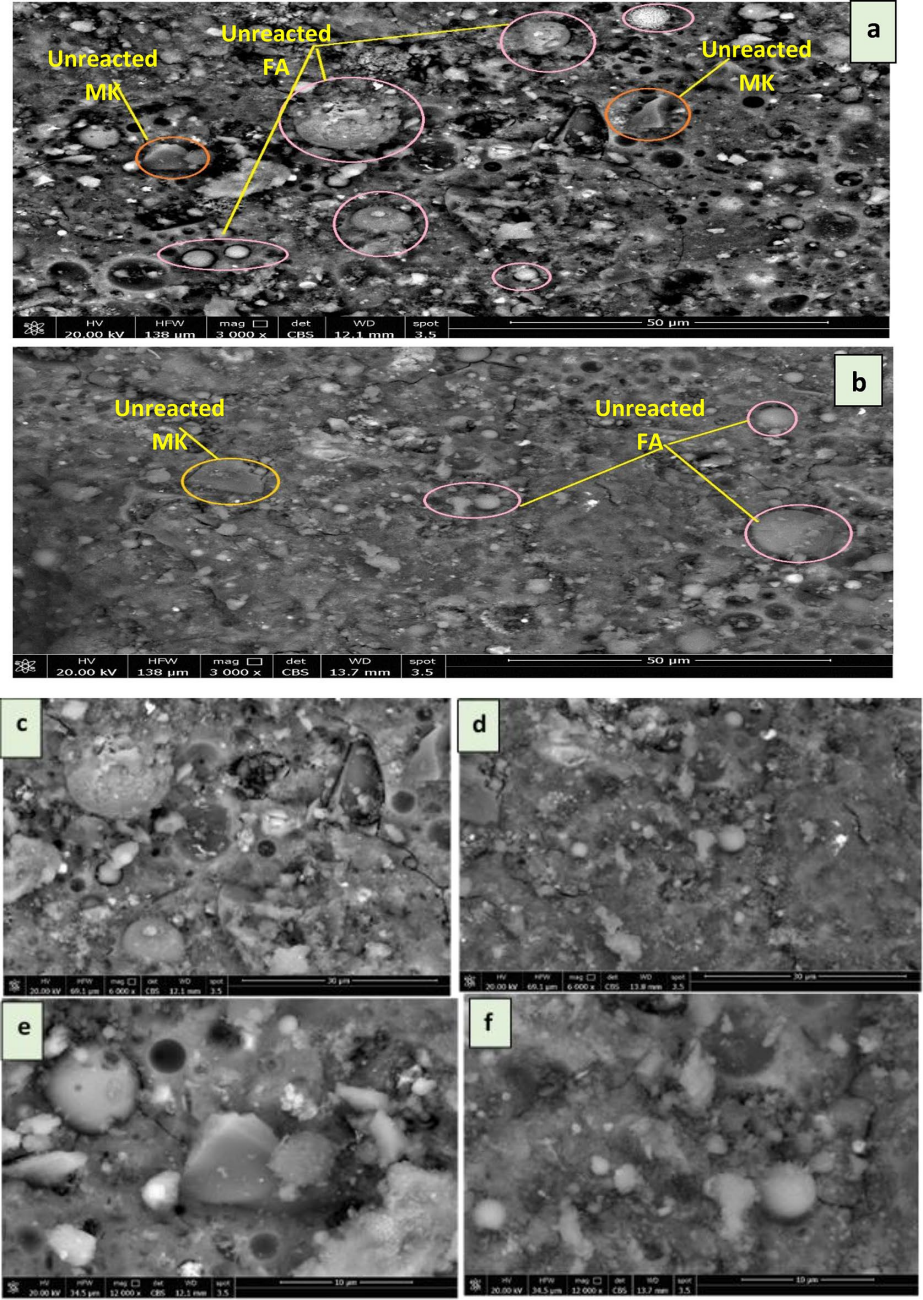
SEM image of geopolymer composite mixes after 7 days of hydration, corresponding different magnification view; (**a**) MF (3000x); (**b**) MF-TiO_2_ (3000x); (**c**) MF (6000 x); (**d**) MF-TiO_2_ (6000x); (**e**) MF (12,000 x); (**f**) MF-TiO_2_ (12000x).

SEM imaging was used to examine the morphology of the geopolymer composite mixes (FM. FM-TiO_2_) after 7 days of hydration, as shown in Fig. 6a,b,c,d,e,f. The microstructure of the FM geopolymer (Fig. 6a) appeared to be less dense and more porous than that of the FM-TiO_2_ geopolymer-NanoTiO_2_ composite mix (Fig. 6b). This could be due to the development of more aluminosilicate gel in the geopolymer composite, resulting in greater compactness^49,56^. Moreover, the evenly dispersed nanoparticles served as fillers in the empty spaces, filling the gaps created by the geopolymer particles^56^. The addition of NT led to densification of the microstructure of geopolymer composites, according to the authors^49^. The nucleation and nanopore-filling effects of NT also influenced the rate of hydration^49^. Figure 6a,b,c,d,e,f shows that the microstructure of the FM-based geopolymer composite was improved by the addition of 2.5% NT particles, resulting in more reacted fly ash and metakaolin particles, as well as a dense structure. Our findings are consistent with previous studies conducted by Samsidar-Nurfadilla and Sahitya -Sastry^57,58^, which showed that the use of NT reduces the size and amount of unreacted FA particles and accelerates saturation resulting in the production of saturated materials through polymerization and nanoparticle stuffing.

### Different applications of FM and FM-TiO_2_ geopolymer composites

#### Antibacterial activity of geopolymer composites

**Figure 7:**
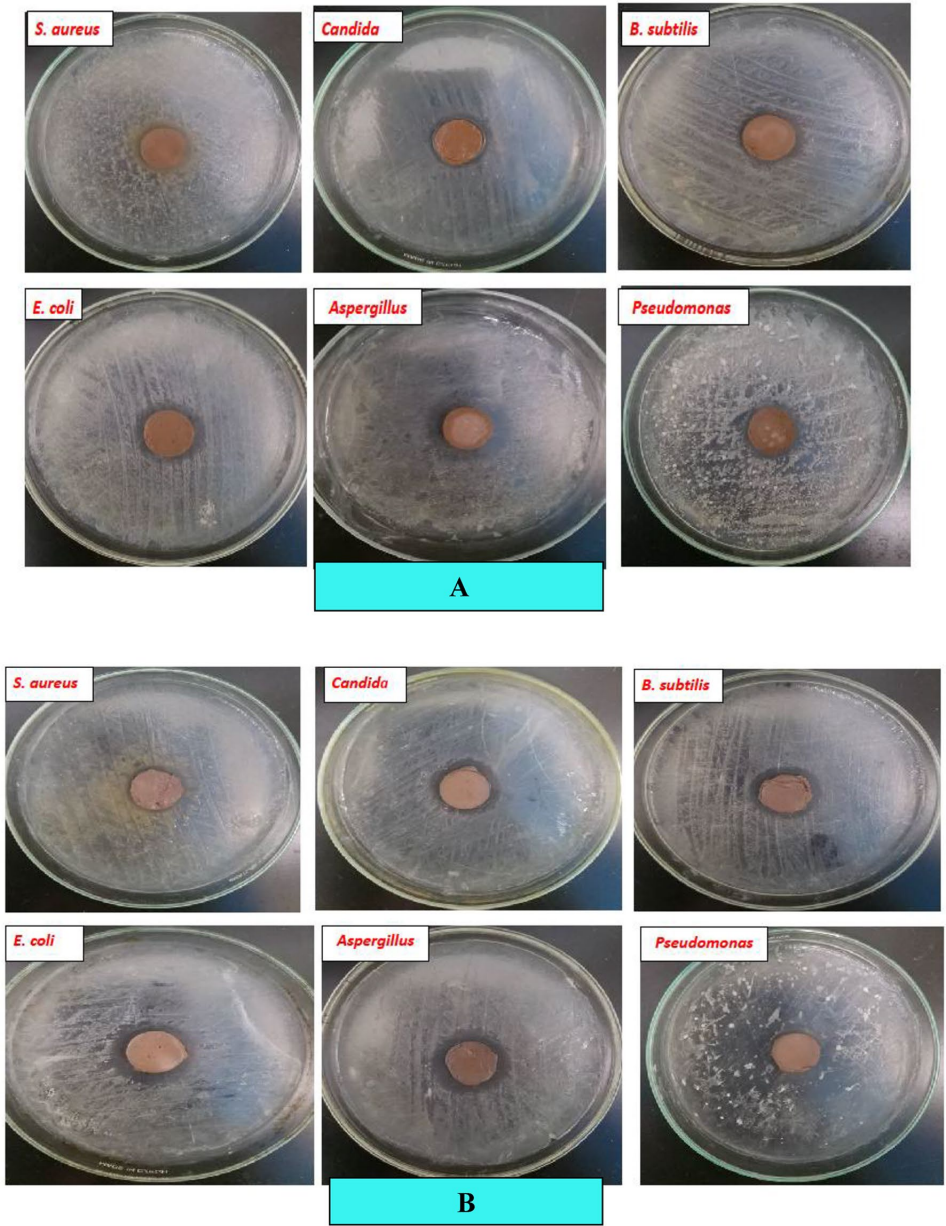
The images display the zone of inhibition of: (**A**) FM, and (**B**) FM-TiO_2_ against several Gram-positive and Gram-negative microorganisms.

First, we study the antibacterial application of our prepared eco-friendly inorganic polymer composites, FM and FM-TiO_2_, towards different microbes. According to Table 3 and Fig. 7A,B, both geopolymer composites with and without nanoparticles generally exhibited significant antibacterial activity, as shown by measured zones of inhibition. The geopolymer sample FM-TiO_2_ has better biological activity for Escherichia coli, Candida albicans, and Pseudomonas aeruginosa than the FM composite. This may be attributed to the fact that the presence of nano-TiO_2_ particles inside the geopolymer matrix enhanced the biological activity of geopolymer materials towards some microorganisms compared to others^34^. These findings supported the outcomes of another recent investigation by Gutiérrez et al., 2020^59^, which found that geopolymers containing TiO_2_ nanoparticles were "satisfactory" in inhibiting the growth of the bacteria E. coli and Pseudomonas aeruginosa. However, the geopolymer FM and FM-TiO_2_ demonstrate the same antibacterial efficacy against Aspergillus niger. According to our results, the addition of nano-TiO_2_ enhanced the antimicrobial activity of the eco-friendly fly ash/metakaolin geopolymer composite.

**Table 3:** The inhibition zone diameter values (in cm) of fly ash/metakaolin based-geopolymer composite with and without Nano-TiO_2_ against different test microorganisms.

Sample name	Microorganisms type					
	*E. Coli*	Aspergillus	Candida	Pseudomonas	*S. Aureus*	*B. Subtilis*
FM	2.20	3.00	2.00	2.30	2.20	2.20
FM-TiO_2_	3.20	3.00	2.20	3.00	2.10	2.20

#### Adsorption and photocatalytic activity of FM and FM-TiO_2_ geopolymer composites

The two new environmentally friendly geopolymers developed in this study can also remove dye effluent from wastewater as another application. It is known that dyes are widely used in dyeing fabrics, especially blue. And when using reactive dyes in dyeing fabrics, especially when using high concentrations, about 40% of the original dye weight remains in the dyeing bath, so we reused the dyeing bath, and yet about 5% of the dyeing weight remains. Therefore, the aim of this part of the study was to treat the wastewater from the dyeing baths in order to reuse it and preserve the environment at the same time.

**Figure 8:**
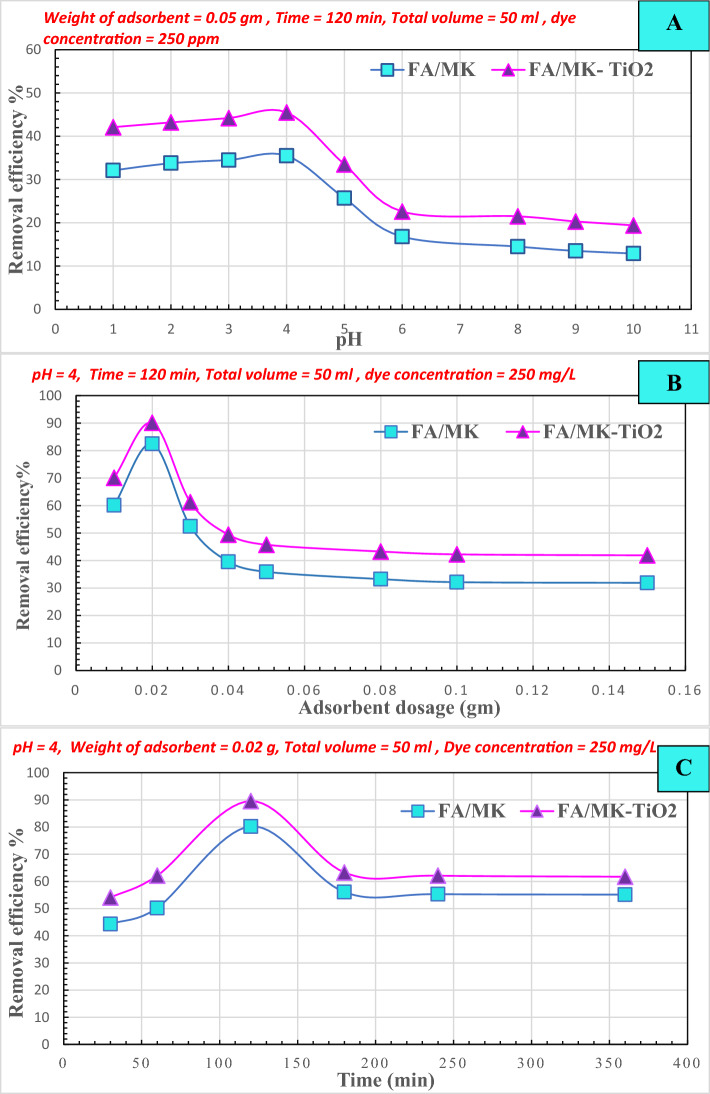
Different adsorption factors affecting on reactive blue 19 dye effluent removal efficiency % by geopolymer composites FM and FM-TiO_2_: (**A**) pH, (**B**) Adsorbent Weight, and (**C**) Time.
  2. Effect of the dosage of adsorbent
    Based on Fig. 8B, the quantity of absorbent has an impact on the removal efficiency. The experiment involved testing the adsorption of dye effluents using reactive blue 19 dye (250 mg/L) and different amounts (ranging from 0.01 to 0.15 g/50 mL) of geopolymer composites (FM and FM-TiO_2_) for 120 min, with a pH of 4. The removal efficiency decreases as the weight of the absorbent increases, as shown in Fig. 8B. At 0.02 g/50 mL, the FM-TiO_2_ geopolymer composite exhibited a maximum removal efficiency of 90.1%, while the FM geopolymer mix achieved the highest removal efficiency of 82.5% at the same absorbent weight.
  3. Effect of time
    The ideal time for reactive blue 19 dye effluent (250 mg/L) interaction with the fly ash/metakaolin geopolymer composite, with or without nano-TiO_2_, was determined by treating the composites under different procedure for varying time periods (30–360 min). The results depicted in Fig. 8C illustrate how prolonging the adsorption period could improve the dye's removal efficiency. The amount of color that was removed improved over time until it reached a maximum value, at which point removal efficiency stabilized. The decolorization rate for the FM geopolymer composite at 120 min is 80.22%, while it is 89.56% for the FM-TiO_2_ geopolymer composite.
    From all the adsorption test results, we observed that the FM-TiO_2_ has a larger adsorption capacity than the FM, which suggests that applying TiO_2_ to the geopolymer may enhance its adsorption capacity^60^. As a result of their porous structures and the presence of active sites on their surfaces, the FM and FM-TiO_2_ composites both exhibit adsorption capabilities^60^.
B. Sunlight photodegradation of reactive blue 19 dye effluent
  In our previous study^13^, we modified a metakaolin /cement kiln dust—based geopolymer composite to remove organic reactive dye effluent from wastewater by two different methods: adsorption and solidification of organic pollutants inside the geopolymer matrix. In this study, we examined the removal of reactive organic dye pollutants by adsorption and photodegradation under sunlight as a low-cost and effective removal method. We investigated the photocatalytic activity of novel fly ash/metakaolin based- geopolymer composite with and without nano-titanium oxide (FM-TiO_2_) against reactive blue 19 dye effluent. Both geopolymer composites were subjected to direct exposure to ultraviolet (UV) radiation from the sunlight in order to conduct the photodegradation test^61^. Figure 9 illustrate the degradation degree % of reactive blue 19 dye effluent solution (50 mg/L) under sunlight using 0.1 g of FM and FM-TiO_2_ geopolymer for a period of 30 min to 6 h. Figure 8 shows also, the effectiveness of geopolymer FM-TiO_2_ in removing reactive blue 19 dye effluent in the absence of light. To determine the amount of dye effluent adsorbed and degraded by geopolymer composites in sunlight, we examined the removal of dye effluent in the dark during the same duration (Table 4). Based on the results presented in Fig. 9 and Table 4, it is evident that the dye effluent solution begins to degrade after being exposed to sunlight for 30 min. As time goes on, the rate of degradation increases, providing evidence of the active role played by the TiO_2_ nanoparticles that are incorporated in the geopolymer paste as photocatalysts. It is worth noting that geopolymer FM on its own exhibits excellent efficiency in removing and degrading reactive blue 19 dye effluent (RB) when exposed to sunlight^61,62^. This can be attributed to the presence of various oxides, such as Fe_2_O_3_, CuO, and TiO_2_, in the geopolymer composition, as evidenced by the XRF analysis of the fly ash and metakaolin raw materials (Table 1). In the first 30 min, all geopolymer composites (FM and FM-TiO_2_) show a rapid decrease in RB dye concentration. This is due to the availability of active sites on the geopolymer composite surface for adsorption, which become saturated over longer adsorption durations^61^. Our study suggests that both the adsorption and degradation mechanisms are involved in RB dye pollutant removal, as indicated in Table 4. According to Fig. 9 and Table 4, both geopolymer composites achieved maximum RB removal in approximately the first 90 min. After that, the adsorption process slowed down, while photodegradation appeared to slightly increase over time. It stabilized after 180 min. The photocatalyst's active sites are deactivated by significant by-product deposition, causing a short lifetime and slow dye degradation after long time^62^.

A. Adsorption efficiency of FM and FM-TiO_2_ geopolymer composites
  1. Effect of pH on the removal efficiency
    The Fig. 8A demonstrates the effect of pH on the removal of reactive blue 19 dye effluent (250 mg/L) from an adsorption bath. Additionally, it was feasible to calculate the optimal pH value for geopolymer composite adsorption by treating dye effluent with fly ash/metakaolin-based geopolymers (FM and FM-TiO_2_) at a variety of pH ranges (2–10). According to the outcomes from Fig. 8A, the percentage of dye removal decreases with a pH higher than 4. Furthermore, the removal efficiencies for reactive blue dye effluents were at their highest levels when using the geopolymer composites FM and FM-TiO_2_, which reached their maximum values at 35.5% and 45.5%, respectively, at pH 4. The optimal pH for the adsorption and photodegradation studies was determined to be 4, as higher pH values decreased dye adsorption. We also observed that the addition of NT particles to the geopolymer increased its adsorption towards reactive blue 19 dye effluent under all pH ranges.

**Figure 9:**
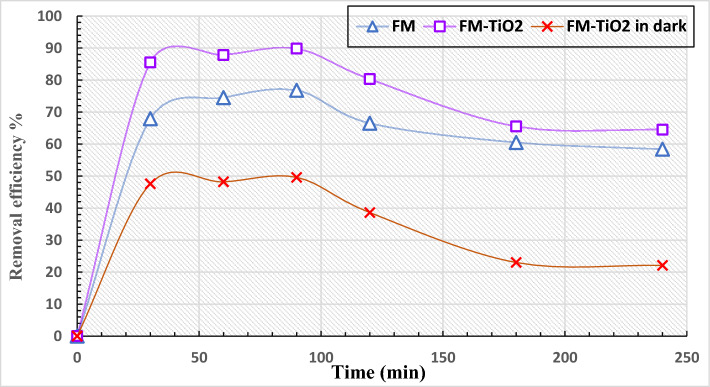
The removal efficiency % of reactive blue 19 dye effluent with FM and FM-TiO_2_ under different time, in dark and after exposure to sunlight. (wt. of adsorbent 0.1 g, Temperature 30 °C, pH 4, concentration of dye 50 mg/L).

**Table 4:** Values of adsorption and degradation % for reactive blue 19 effluent loaded by nano-TiO_2_-FM geopolymer composites after different time under sunlight and dark condition.

Geopolymer mix	Removal efficiency %					
	30 min	60 min	90 min	120 min	180 min	240 min
FM-TiO_2_ (Sunlight)	86.5	87.8	89.8	80.3	65.5	64.5
FM-TiO_2_ (Dark)	47.6	48.2	49.6	38.6	23.0	22.1
Adsorption %	47.6	48.2	49.6	38.6	23.0	22.1
Degradation %	38.9	39.6	40.2	41.7	42.5	42.4

Significant are in value [bold].

## Conclusion

Our study results reveal that novel geopolymer composites made of 70% fly ash and 30% metakaolin (FM) offer sustainable, eco-friendly alternatives to conventional cement. The outcomes also show that adding 2.5% nano-TiO_2_ particles to geopolymer composite, FM, has a beneficial impact on its mechanical and antibacterial properties as well as in the environment application for removing industrial organic dye contaminants from wastewater. The following conclusions can be made in light of the experimental findings:

- The compressive strength of geopolymer composite FM is improved when 2.5 wt.% of nano-TiO_2_ (FM-TiO_2_) is added at all hydration periods, from 3 to 28 days.
- The addition of nano-TiO_2_ to geopolymer composite MF results in a reduction in total porosity values at all hydration ages, which can be attributed to inter-particle diffusion of nano-TiO_2_ into the internal pores of the geopolymer matrix, leading to a decrease in the total number of pores.
- The XRD patterns and SEM images suggest that the inclusion of nano-TiO_2_ fillers in the geopolymer composites accelerate the geopolymerization process. This is evidenced by the decrease in size and quantity of unreacted raw material and the densification of the microstructure of geopolymer composites FM.
- The FTIR spectra for geopolymer composites FM and FM-TiO_2_ indicate that excessive hydration products may develop and precipitate inside the geopolymer matrix. However, the type of hydration products generated did not change significantly upon the addition of NT.
- Both FM and FM-TiO_2_ demonstrated strong antibacterial activity, but FM-TiO_2_ showed superior biological activity against Escherichia coli, Candida albicans, and Pseudomonas aeruginosa compared to FM.
- Based on photodegradation data, it has been found that the effluent of reactive blue 19 dye (RB) can be effectively removed and degraded by both the geopolymers FM and FM-TiO_2_ when exposed to sunlight. But presence of NT improve the reactive blue 19 dye degradation.

This research holds significant importance in the development of eco-friendly and sustainable construction materials that possess antibacterial properties and can efficiently eliminate harmful pollutants from wastewater. The use of such materials has the potential to revolutionize the construction industry by addressing both environmental and health-related concerns.

**Figure 10:**
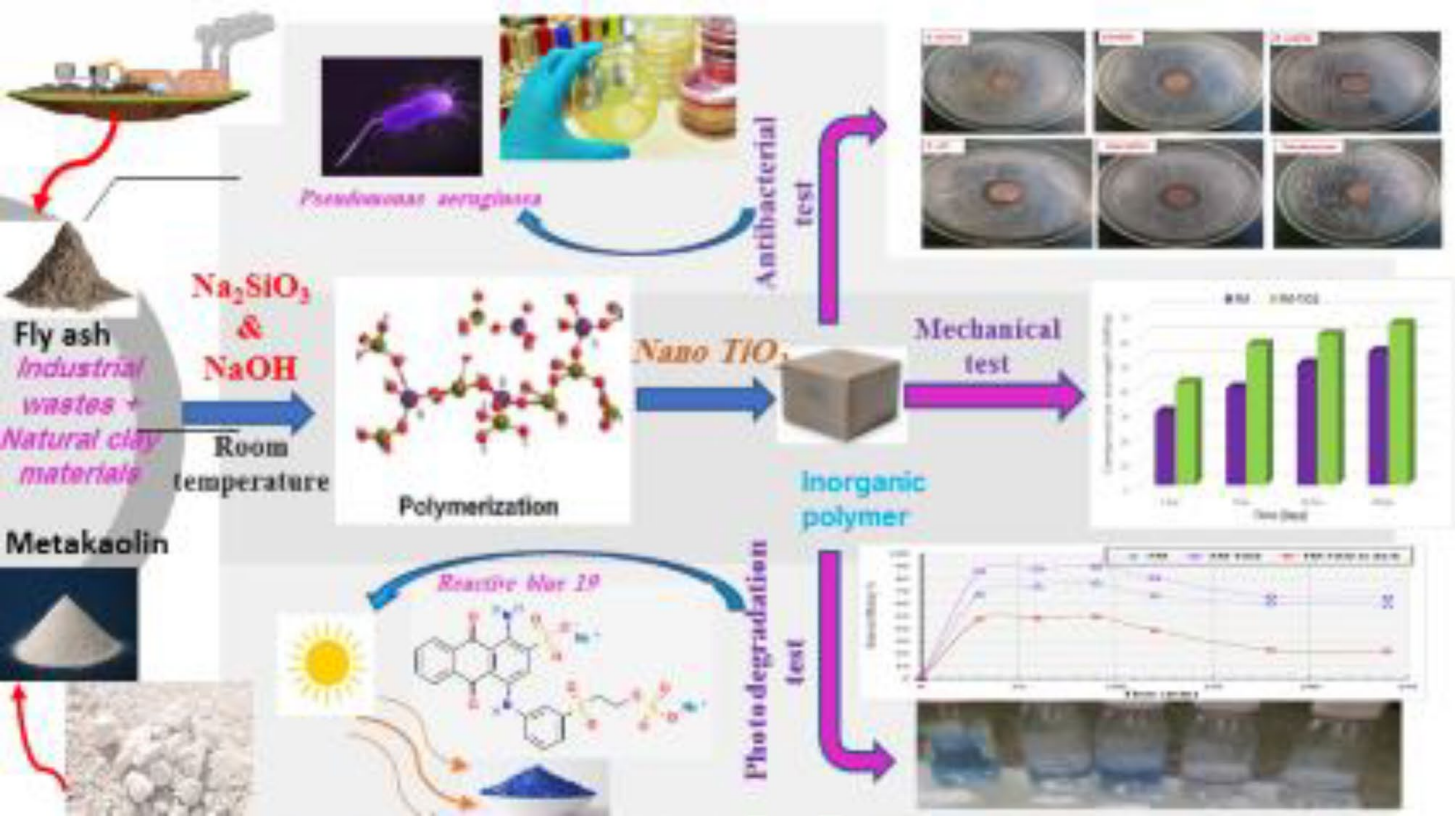
Diagram illustrating our study’s targets.

Figure 10 depicts the main goals of our investigation.

## Data Availability

All data generated or analyzed during this study are included in this published article. Datasets are available in the manuscript.
